# Supplementary material for: Air mass source determines airborne microbial diversity at the ocean–atmosphere interface of the Great Barrier Reef marine ecosystem
Source: ISME J. 2019 Nov 21;14(3):871–6. doi: 10.1038/s41396-019-0555-0 (PMC7031240; doi:10.1038/s41396-019-0555-0)
Supplement: Supplementary file 1 — supplementary Information [file 41396_2019_555_MOESM1_ESM.pdf]

Air mass source determines airborne microbial diversity at the ocean-atmosphere interface of the Great Barrier Reef marine ecosystem

Stephen D.J. Archer, Kevin C Lee, Tancredi Caruso, Katie King-Miaow, Mike Harvey, Danwei Huang, Benjamin J Wainwright, Stephen B Pointing

Supplementary Information

Supplementary Methods

Sample Recovery

Our study took advantage of a persistent flat-calm sea state between 30th September to 22nd October 2016 (www.marineweather.net.au) that minimised interference from microorganisms that are aerosolised by marine spray in heavier sea states at the Great Barrier Reef. The voyage of the *RV Investigator* circumnavigated the reef. We sampled air mass daily from the deck of the research vessel approximately 25m above the sea surface. Massive bulk-phase air volumes (18-108 m^3 per sample) were collected for each discrete sampling station. Overall, we retrieved 53 independent bulk-phase air samples (Supplementary Information, Table S1).

We employed a high-volume liquid impinger apparatus (Coriolis μ, Bertin Technologies, France) and collected aerosol particulate fractions directly into phosphate-buffered saline (PBS) using collection cones supplied by the manufacturer. Evaporation was compensated for using a peristaltic pump set to between 0.5 and 1.3 mL per minute with PBS. Random collection cones were also assembled into the machine but not activated, and these were used as the negative controls. All collection cones were soaked in 1.5 % sodium hypochlorite (NaClO) then washed with 70 % ethanol and three washes of Milli-Q H_20 before being filled with filtered PBS. All sampling equipment was disassembled between locations and cleaned with NaClO, ethanol and Milli-Q H_20. Samples were processed immediately on board the vessel.

Back-trajectories of air mass arriving at each sampling interval were generated using the National Oceanic and Atmospheric Administration (NOAA) HYSPLIT-WEB model (https://ready.arl.noaa.gov/HYSPLIT.php) (Fig. S1). Calculations were made with a 3 day back trajectory because this is estimated as the average residence time for microbial cells in air [1] (Fig. S1). We acknowledge that large variation is inherent in

models of atmospheric transport and uncertainty increases with duration of the back trajectory estimate. With our model the estimated trajectories greater than two weeks resulted in diffuse origins for all samples from above the Southern Ocean. The HYSPLIT back trajectories were calculated using the GDAS database and the model vertical velocity option. The continental or oceanic origin of air mass was further verified by measuring the concurrent concentration of atmospheric radon gas on board the research vessel in real time by the Australian Nuclear Science and Technology Organisation (ANSTO) (Supplementary Information, Table S1) [2], with the hypothesis that radon levels are typically higher from back trajectories originating over continental Australia compared with those that originate from the ocean. Radon is emitted only from unsaturated, unfrozen terrestrial surfaces and has a half-life of 3.8 days, making it a powerful tracer of continental air transport and mixing.

Environmental DNA sequencing and bioinformatics

Airborne microbial samples suspended in PBS were filtered onto a 25 mm 0.2 μm polycarbonate filter and stored frozen until processed. Total DNA was directly extracted using a CTAB protocol [3]. DNA yield for these ultra-low biomass samples was quantified using the Qubit 2.0 Fluorometer (Invitrogen) (Supplementary Information, Table S1). Samples were then stored at -20 °C until processed. We used DNA yield as an indirect estimate for biomass.

Illumina MiSeq libraries were prepared as per manufacturer's protocol (Metagenomic Sequencing Library Preparation Part # 15044223 Rev. B; Illumina, San Diego, CA, USA) and as previously described with PhiX positive controls [4]. We targeted bacteria and fungi since these domains are the most abundant microorganisms in aerosols [5]. PCR was conducted with primer sets targeting the V3-V4 regions of

bacterial and archaeal 16S rRNA gene: PCR1 forward (5' TCGTCGGCAG CGTCAGATGT GTATAAGAGA CAGCCTACGG GNGGCWGCAG 3') and PCR1 reverse (5' GTCTCGTGGG CTCGGAGATG TGTATAAGAG ACAGGACTAC HVGGGTATCT AATCC 3') and the internal transcribed spacer region of fungal 18S and 5.8S rRNA genes: ITS1 forward (5' CTTGGTCATTTAGAGGAAGTAA 3') and ITS2 reverse (5' GCTGCGTTCTTCATCGATGC 3'). These primers for Bacteria and Fungi are widely accepted to capture the broadest estimates of diversity [6–8]. Total sequence library sizes were 12,027,554 for bacteria and 8,570,900 for fungi before filtering. Total counts in the processed dataset were 4,727,038 bacterial and 2,969,686 fungal sequences. A total of 1386 bacterial and 3775 fungal taxa were identified from these.

Sequencing data for bacterial 16S rRNA gene and fungal ITS1 amplicons was processed based on the DADA2 v1.8 [9] pipeline. Primers sequences were removed using cutadapt [10] to remove forward (CCTACGGGNGGCWGCAG) and reverse (GACTACHVGGGTATCTAATCC) for 16S rRNA gene, and forward (CTTGGTCATTTAGAGGAAGTAA) and reverse (CTTGGTCATTTAGAGGAAGTAA) for fungal ITS1 region. Bacterial reads were uniformly trimmed to 280 bp (forward) and 250 bp (reverse) and then filtered by removing reads exceeding maximum expected error of 2 for forward reads and 5 for reverse reads or reads containing ambiguity N symbol. Fungal reads were untrimmed (due length hyper-variability of the region) and reads exceeding maximum expected error of 5 for forward reads and 8 for reverse reads, or those with ambiguity N symbols were removed. The reads were used to train the error model and then dereplicated to acquire unique sequences, which were used to infer sequence variants with the trained error model. The forward and reverse reads were merged and chimeric sequences were removed. For bacteria we used amplicon sequence variants (ASVs) to assign taxa since this has been shown as the most robust

method currently available for bacterial 16S rRNA gene-defined taxa identification [11]. ASVs were given taxonomic assignment using DADA2 with SILVA nr v132 database [12] for bacterial reads to provide species level assignment based on exact match between ASVs and known reference sequences. . Fungal reads were taxonomically classified using UNITE v7.2 database [13]. All sequence data generated by this study has been submitted to the EMBL European Nucleotide Archive (ENL) under BioProject PRJEB31630 with sample accession numbers ERS3215240 to ERS3215312.

The resulting taxa were then processed as previously described [14] [15]. The R packages phyloseq [16], DESeq2 [17] and ggplot2 [18] were used for downstream analysis and visualisation including ordination and alpha/beta diversity calculations. For heatmap visualisations the 1,000 most abundant ASVs in each dataset were selected and these captured 99.99 % bacterial and 91.74 % fungal reads in the libraries. All other analyses used the entire sequence library data. Our heatmap analysis therefore had high confidence since the unsampled 'tail' comprised only extremely rare sequence variants at very low/singleton abundance.

Sequencing of control samples revealed very low recovery of putative contaminant microbial signatures, a total of only 17 out of 1403 bacterial and 5 out of 3775 fungal sequences were statistically classified as putative contaminants with *decontam* 1.2.0 [19].

Meta-analysis of atmospheric microbiome with coral microbiomes

A meta-analysis of our data with publicly available sequence data from coral-associated microbiomes was conducted. We first searched for relevant recent published studies and constrained our search as follows: The first 20 matches by relevance from the search parameters ["16S" "coral"] and ["16S" "reef"] from 2015 to 2019 with Google

Scholar were employed to determine if there was any publicly available next-generation sequencing data that encompassed the compatible 16S rRNA gene region. Based on these criteria, we identified a single publication [20] which allowed for direct ASV analysis. Another dataset with limited overlap was also assessed based on similarity searches of representative sequences to determine shared taxa [21]. Some excellent studies on Great Barrier Reef coral microbiomes could not be included because they have employed the V1-V3 region of the 16S rRNA gene for bacteria and this does not overlap with the V3-V4 region used in our study. We also searched nucleotide databases (NCBI and EMBL) for unpublished but publicly available coral microbiome sequences using the same search criteria. This search revealed three entries in the top 50 matches by relevance but our initial quality checking revealed these were unsuitable for further inclusion in our analysis.

PacBio SMRT sequencing of 16S rRNA gene amplicons from corals of Gulf of Thailand and Andaman Sea was conducted by Pootakham et al., (2017)[20]. The raw reads were retrieved from NCBI Short Read Archive (accession SRR5149735) via NCBI SRA toolkit (https://github.com/ncbi/sra-tools/wiki). As the reads from this study extend beyond those of our study (V3-V4), we were able to process the reads in a manner similar to our study and trim the reads based on the primer regions of our study (341F/785R). This resulted in reads targeting homologous regions for comparison between the two studies. The trimmed reads followed a similar processing pipeline as our study (dada2 for ASV-calling and taxonomic classification, see below), which enabled direct and precise comparison of ASV sequences.

The Wainwright et al., (2019) study [21] conducted paired-end Illumina sequencing of 16S rRNA gene amplicons of the coral *Pocillopora acuta* around Singapore. The coral study utilised comparable data processing pipeline (specifically

dada2 to generate ASV) but used a different set of primers to amplify 16S rRNA gene (V4 vs our V3-V4 region). However, a significant portion of the partial 16S rRNA gene amplicon still overlapped (~250 bp). The ASVs from our study were searched against reference ASVs(https://github.com/gzahn/Pocillopora_Bacteria) from Wainwright et al. (2019) using the usearch v10.0.240_i86linux32 global function [22]. Both strand orientations were searched (-strand both) and a minimum identity threshold (-id) of 0.97 was set. The ASVs were sorted with descending number in relation to mean relative abundance (i.e. ASV1 has highest mean relative abundance).

We also conducted a more relaxed analysis where the 100 most abundant bacterial and fungal taxa at genus level (Table S1-3) were compared to published studies that used different approaches for coral microbiome diversity estimation [23–35]. Microorganisms unassigned at the Genus taxonomic level were excluded from further analysis. A weighted average of the relative abundance was calculated for each ASV identified in this study, grouping samples with an ocean air mass source, and a continental air mass source. Near full length 16S rRNA gene sequences of coral associated bacteria were searched against our ASV sequences using usearch_local function in usearch v10.0.240 to identify highly similar matches.

For the fungi no comparable sequence data for coral-associated fungi is available. We therefore performed an approximate comparison at the Genus level with reference to published descriptions of coral-associated fungi [23, 24, 28, 29]. There are no bacterial or fungal taxa that have yet been unequivocally been identified as either obligate symbionts [27] or pathogens [36] of corals.

Statistical Treatments

Community phylogenetic metrics including were calculated using the R [37] packages picante [38], ape [39], phylobase [40], adephylo [41], and phytools [42]. We tested partitioning of Bray-Curtis dissimilarity matrices by air mass origins (continent or ocean) through PERMANOVA, using the *adonis* function from R package vegan 2.5-5 [43] with 999 unrestricted permutations. Phylogenetic trees for community phylogenetic structure analysis were constructed for all OTUs with FastTree v2.1.9 on multiple alignment of sequences produced by MUSCLE v3.8.31. For Bacteria an approximately Maximum-Likelihood approach was used whilst for Fungi an alignment-free distance approach with Neighbour-Joining method was employed in order to generate a ITS-based phylogenetic tree for community metrics [44]. The distance approach was used for Fungi as the hypervariability of ITS1 loci hinders multiple sequence alignment required for most phylogenetic analyses. To validate this approach, we compared our tree topology with the most recent whole genome phylogenies for the Fungi [45, 46]. This approach was robust at higher taxonomic levels (there is no consensus for fungal phylogenies using multiple loci or whole genomes below Order rank) and has been used successfully with other eukaryotic taxa as a workflow for Net Relatedness analysis [47]. Although we acknowledge limitations to this approach, the advantages of using ITS loci for taxonomic identification vastly outweighed its shortcomings, and we also triangulated data from this test with additional analytical approaches, so overall our inclusion of this test is justified and interpreted conservatively. Mean phylogenetic distance (MPD) [48] was calculated to measure phylogenetic distance between ASVs and OTUs in each sample. A null model algorithm based on independent swap (999 randomisations) was used to test the extent of

phylogenetically clustering (positive values for the NRI effect size) or over-dispersion (negative values for the NRI effect size) [49].

Methods References

1. Burrows SM, Butler T, Jöckel P, Tost H, Kerkweg A, Pöschl U, et al. Bacteria in the global atmosphere – Part 2: Modeling of emissions and transport between different ecosystems. *Atmos Chem Phys* 2009; **9**: 9281–9297.
2. Chambers SD, Williams AG, Crawford J, Griffiths AD. On the use of radon for quantifying the effects of atmospheric stability on urban emissions. *Atmos Chem Phys* 2015; **15**: 1175–1190.
3. Archer SDJ, McDonald IR, Herbold CW, Lee CK, Cary CS. Benthic microbial communities of coastal terrestrial and ice shelf Antarctic meltwater ponds. *Front Microbiol* . 2015. , **6**: 485
4. Warren-Rhodes K, Lee K, Archer S, Cabrol N, Ng-Boyle L, Wettergreen D, et al. Subsurface microbial habitats in an extreme desert Mars-analogue environment. *Front Microbiol* 2019; **10**: 10.3389/fmicb.2019.00069.
5. Mayol E, Arrieta JM, Jiménez MA, Martínez-Asensio A, Garcias-Bonet N, Dachs J, et al. Long-range transport of airborne microbes over the global tropical and subtropical ocean. *Nat Commun* 2017; **8**: 201.
6. Schoch CL, Seifert KA, Huhndorf S, Robert V, Spouge JL, Levesque CA, et al. Nuclear ribosomal internal transcribed spacer (ITS) region as a universal DNA barcode marker for Fungi. *Proc Natl Acad Sci* 2012; **109**: 6241–6246.
7. Thompson LR, Sanders JG, McDonald D, Amir A, Ladau J, Locey KJ, et al. A communal catalogue reveals Earth’s multiscale microbial diversity. *Nature* 2017; **551**: 457–463.

8. Delgado-Baquerizo M, Oliverio AM, Brewer TE, Benavent-González A, Eldridge DJ, Bardgett RD, et al. A global atlas of the dominant bacteria found in soil. *Science (80-)* 2018; **359**: 320–325.

9. Callahan BJ, Mcmurdie PJ, Rosen MJ, Han AW, A AJ. DADA2: High resolution sample inference from Illumina amplicon data. *Nat Methods* 2016; **13**: 581–583.

10. Martin M. Cutadapt removes adapter sequences from high-throughput sequencing reads. *EMBnet.journal* 2011; **17**: 10.

11. Callahan BJ, McMurdie PJ, Holmes SP. Exact sequence variants should replace operational taxonomic units in marker-gene data analysis. *ISME J* 2017; **11**: 2639–2643.

12. Quast C, Pruesse E, Yilmaz P, Gerken J, Schweer T, Yarza P, et al. The SILVA ribosomal RNA gene database project: Improved data processing and web-based tools. *Nucleic Acids Res* 2013; **41**: 590–596.

13. Nilsson RH, Larsson K-H, Taylor AFS, Bengtsson-Palme J, Jeppesen TS, Schigel D, et al. The UNITE database for molecular identification of fungi: handling dark taxa and parallel taxonomic classifications. *Nucleic Acids Res* 2019; **47**: D259–D264.

14. Maki T, Kurosaki Y, Onishi K, Lee KC, Pointing SB, Jugder D, et al. Variations in the structure of airborne bacterial communities in Tsogt-Ovoo of Gobi desert area during dust events. *Air Qual Atmos Heal* 2017; **10**: 249–260.

15. Faith DP. Conservation evaluation and phylogenetic diversity. *Biol Conserv* 1992; **61**: 1–10.

16. McMurdie PJ, Holmes S. phyloseq: An R Package for Reproducible Interactive Analysis and Graphics of Microbiome Census Data. *PLoS One* 2013; **8**: e61217.

17. Love MI, Huber W, Anders S. Moderated estimation of fold change and dispersion for RNA-seq data with DESeq2. *Genome Biol* 2014; **15**: 550.

18. Wickham H. ggplot2. 2009. Springer New York, New York, NY.

19. Davis NM, Proctor DM, Holmes SP, Relman DA, Callahan BJ. Simple statistical identification and removal of contaminant sequences in marker-gene and metagenomics data. *Microbiome* 2018; **6**: 226.

20. Pootakham W, Mhuantong W, Yoocha T, Putchim L, Sonthirod C, Naktang C, et al. High resolution profiling of coral-associated bacterial communities using full-length 16S rRNA sequence data from PacBio SMRT sequencing system. *Sci Rep* 2017; **7**: 2774.

21. Wainwright BJ, Afiq-Rosli L, Zahn GL, Huang D. Characterisation of coral-associated bacterial communities in an urbanised marine environment shows strong divergence over small geographic scales. *Coral Reefs* 2019; https://doi.org/10.1007/s00338-019-01837-1.

22. Edgar RC. Search and clustering orders of magnitude faster than BLAST. *Bioinformatics* 2010; **26**: 2460–2461.

23. Littman R, Willis BL, Bourne DG. Metagenomic analysis of the coral holobiont during a natural bleaching event on the Great Barrier Reef. *Environ Microbiol Rep* 2011; **3**: 651–660.

24. Morrison-Gardiner S. Dominant fungi from Australian coral reefs. *Fungal Divers* 2002; **9**: 105–121.

25. Ainsworth TD, Krause L, Bridge T, Torda G, Raina JB, Zakrzewski M, et al. The coral core microbiome identifies rare bacterial taxa as ubiquitous endosymbionts. *ISME J* 2015; **9**: 2261–2274.

26. Palumbi SR, Ziegler M, Voolstra CR, Yum LK, Seneca FO, Yum LK, et al. Bacterial community dynamics are linked to patterns of coral heat tolerance. *Nat Commun* 2017; **8**.

27. Hernandez-Agreda A, Leggat W, Bongaerts P, Ainsworth TD. The microbial signature provides insight into the mechanistic basis of coral success across reef habitats. *MBio* 2016; **7**: 1–10.

28. Sweet M, Burn D, Croquer A, Leary P. Characterisation of the Bacterial and Fungal Communities Associated with Different Lesion Sizes of Dark Spot Syndrome Occurring in the Coral Stephanocoenia intersepta. *PLoS One* 2013; **8**: 1–9.

29. Sadowsky MJ, Jones PR, Sinigalliano CD, Chun CL, Staley C, Gidley ML, et al. Differential Impacts of Land-Based Sources of Pollution on the Microbiota of Southeast Florida Coral Reefs. *Appl Environ Microbiol* 2017; **83**: 1–16.

30. Kvennefors ECE, Sampayo E, Ridgway T, Barnes AC, Hoegh-Guldberg O. Bacterial Communities of Two Ubiquitous Great Barrier Reef Corals Reveals Both Site- and Species-Specificity of Common Bacterial Associates. *PLoS One* 2010; **5**: e10401.

31. Morgan TC, Tyson GW, Heath C, Rich V, Schaffelke B, Bourne DG, et al. Marine microbial communities of the Great Barrier Reef lagoon are influenced by riverine floodwaters and seasonal weather events. *PeerJ* 2016; **4**: e1511.

32. Bourne DG, Munn CB. Diversity of bacteria associated with the coral Pocillopora damicornis from the Great Barrier Reef. *Environ Microbiol* 2005; **7**: 1162–1174.

33. Cardinale M, Brusetti L, Quatrini P, Borin S, Puglia AM, Rizzi A, et al. Comparison of different primer sets for use in automated ribosomal intergenic spacer analysis of complex bacterial communities. *Appl Environ Microbiol* 2004; **70**: 6147–6156.

34. Sato Y, Willis BL, Bourne DG. Successional changes in bacterial communities during the development of black band disease on the reef coral , Montipora hispida. *ISME J* 2009; **4**: 203–214.

35. Nelson CE, Alldredge AL, Mccliment EA, Amaral-zettler LA, Carlson CA. Depleted dissolved organic carbon and distinct bacterial communities in the water column

of a rapid-flushing coral reef ecosystem. *ISME J* 2011; **5**: 1374–1387.

36. Rosenberg E, Koren O, Reshef L, Efrony R, Zilber-Rosenberg I. The role of microorganisms in coral health, disease and evolution. *Nat Rev Microbiol* 2007; **5**: 355–362.

37. Swenson NG. Functional and Phylogenetic Ecology in R. 2014. Springer New York, New York, NY.

38. Kembel SW, Cowan PD, Helmus MR, Cornwell WK, Morlon H, Ackerly DD, et al. Picante: R tools for integrating phylogenies and ecology. *Bioinformatics* 2010; **26**: 1463–1464.

39. Paradis E. Ape package. https://www.rdocumentation.org/packages/ape/versions/5.2. Accessed 24 Oct 2018.

40. Michonneau F. Phylobase package. https://www.rdocumentation.org/packages/phylobase/versions/0.8.4. Accessed 24 Oct 2018.

41. Dray S. Adephylo package. https://www.rdocumentation.org/packages/adephylo/versions/1.1-11. Accessed 24 Oct 2018.

42. Zhang J. Phylotools package. https://www.rdocumentation.org/packages/phylotools/versions/0.2.2. Accessed 24 Oct 2018.

43. Dixon P. VEGAN, a package of R functions for community ecology. *J Veg Sci* 2003; **14**: 927–930.

44. Höhl M, Rigoutsos I, Ragan MA. Pattern-based phylogenetic distance estimation and tree reconstruction. *Evol Bioinform Online* 2007; **2**: 359–75.

45. Choi J, Kim S-H. A genome Tree of Life for the Fungi kingdom. *Proc Natl Acad Sci U S A* 2017; **114**: 9391–9396.

46. Ebersberger I, de Matos Simoes R, Kupczok A, Gube M, Kothe E, Voigt K, et al. A Consistent Phylogenetic Backbone for the Fungi. *Mol Biol Evol* 2012; **29**: 1319–1334.

47. Boyle EE, Adamowicz SJ. Community phylogenetics: Assessing tree reconstruction methods and the utility of DNA barcodes. *PLoS One* 2015; **10**.

48. Kembel SW, Hubbell SP. The phylogenetic structure of a neotropical forest tree community. *Ecology* 2006; **87**: 86–99.

49. Webb CO, Ackerly DD, Kembel SW. Phylocom: software for the analysis of phylogenetic community structure and trait evolution. *Bioinformatics* 2008; **24**: 2098–2100.

Figure S1. HYSPLIT back trajectory analysis for modelled transit routes with sampling date indicated (full 3 day back trajectories are shown in Fig. 1).

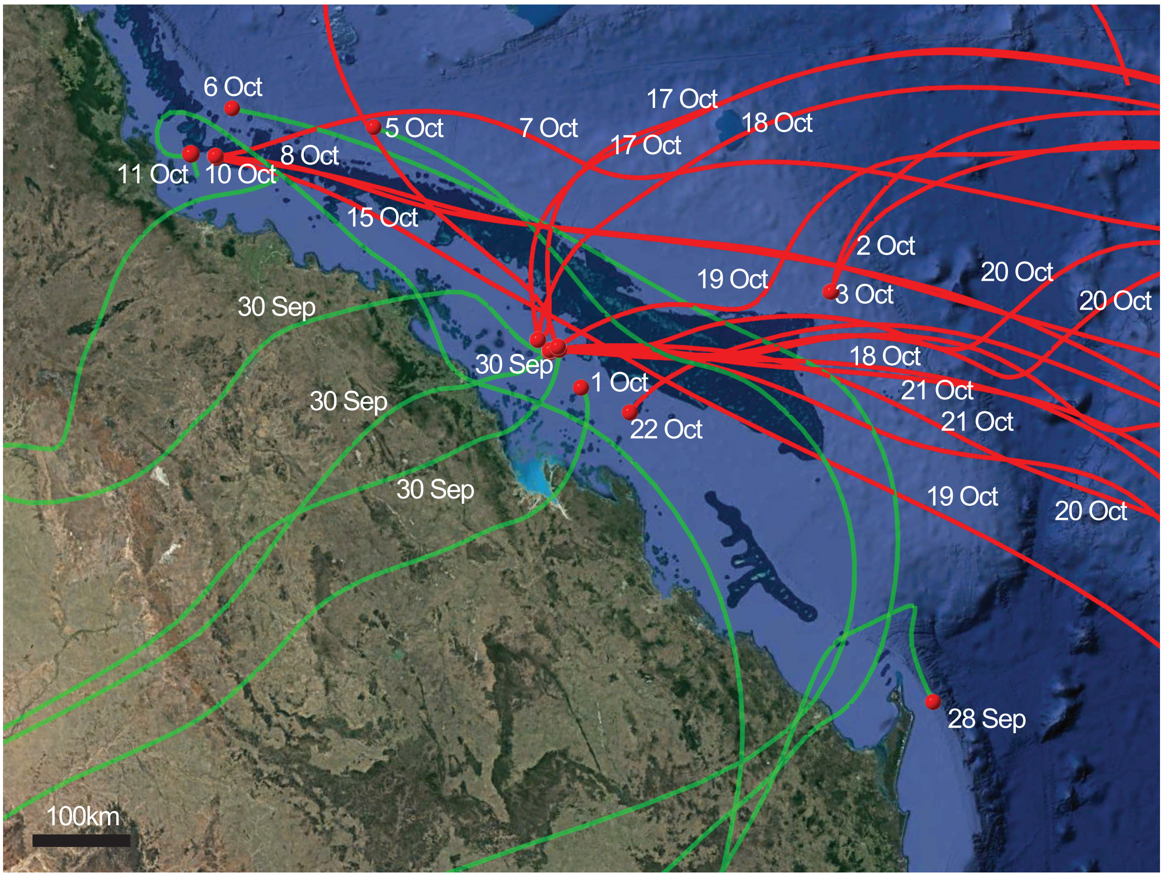

Table S1. Sampling meta-data.

SAMPLE NAME	Long. (decimal degrees)	Lat. (decimal degrees)	Radon (mBq/m^3)	Air Source	Date Start	Time Start	Duration (hrs)	Volume (M^3)	Total DNA ng/M^3	Passed Sequencing (Y/N)
**GBR-01-SA**	24.8973	153.5982	1086.1301	continent	28.09.16	1500	4	72	below detection	Y
**GBR-02-SA**	na	na	na	na	29.09.16	1000	4	72	0.075	N
**GBR-03-1-SA**	-20.9937	150.1999	error	ocean	30.09.16	0800	0.5	9	0.244	Y
**GBR-04-SA**	na	na	na	na	30.09.16	1100	4	72	0.722	N
**GBR-05-1**	-20.9984	150.2017	error	continent	30.09.16	1500	4	72	below detection	Y
**GBR-05-2**	-20.9947	150.2022	error	continent	30.09.16	1900	4	72	0.037	Y
**GBR-05-3**	-21.0264	150.2081	error	continent	30.09.16	2300	4	72	below detection	Y
**-VE CONTROL 1**	na	na	na	control				0	below detection	Control
**GBR-05-4**	21.4199	150.3747	1549.0931	continent	01.10.16	0300	4	72	below detection	Y
**GBR-05-5**	na	na	na	na	01.10.16	0700	4	72	1.467	N
**GBR-05-6**	na	na	na	na	01.10.16	1100	4	72	0.111	N
**GBR-06-1**	na	na	na	na	2.10.16	0730	6	108	below detection	N
**GBR-06-2**	na	na	na	na	2.10.16	1330	6	108	below detection	N
**GBR-06-3**	-20.8275	153.0668	499.6199	ocean	2.10.16	1930	6	108	below detection	Y
**GBR-06-4**	-20.8251	153.0786	552.5687	ocean	3.10.16	0130	2	36	0.199	Y
**-VE CONTROL 2**	na	na	na	control				0	below detection	Control
**GBR-07-SA**	-18.5789	148.6524	904.2033	continent	5.10.16	0900	6	108	0.118	Y
**GBR-08-SA**	-18.1407	147.2207	434.4520	continent	6.10.16	0900	6	108	0.081	Y
**GBR-09-SA**	-18.5786	146.9731	389.6492	ocean	7.10.16	0900	6	108	below detection	Y
**GBR-09-2**	na	na	na	na	7.10.16	1500	6	108	below detection	N
**GBR-10-SA**	18.5847	146.9712	210.4377	ocean	8.10.16	0900	6	108	below detection	Y
**GBR-11-1**	na	na	na	na	9.10.16	0900	6	108	below detection	N
**GBR-11-2**	na	na	na	na	9.10.16	1500	6	108	below detection	N
**-VE CONTROL 3**	na	na	na	control				0	below detection	Control
**GBR-11-3**	na	na	na	na	9.10.16	2100	6	108	0.070	N
**GBR-11-4**	18.5822	146.9790	205.0071	ocean	10.10.16	0300	6	108	0.046	Y
**GBR-11-5**	na	na	na	na	10.10.16	0900	6	108	0.039	N
**GBR-12-SA**	18.5164	146.7233	811.8823	continent	11.10.16	0900	6	108	0.039	Y
**GBR-13-SA**	na	na	na	na	12.10.16	0900	6	108	below detection	N
**GBR-13-2**	na	na	na	na	12.10.16	1500	6	108	below detection	N
**GBR-13-3**	na	na	na	na	13.10.16	0230	3.5	63	below detection	N
**-VE CONTROL 4**	na	na	na	control				0	below detection	Control
**GBR-14-SA**	18.6035	146.9800	279.6785	ocean	15.10.16	1300	4	72	0.028	Y
**GBR-15-1**	20.9020	150.0082	270.1749	na	17.10.16	1400	1	18	below detection	N
**GBR-15-2**	na	na	na	na	17.10.16	1800	1	18	below detection	N
**GBR-15-3**	20.9019	150.0029	268.8172	na	17.10.16	2200	1	18	below detection	N
**GBR-15-4**	na	na	na	na	18.10.16	0200	1	18	below detection	N
**GBR-15-5**	20.8992	150.0069	243.0216	na	18.10.16	0600	1	18	below detection	N

**GBR-15-6**	na	na	na	na	18.10.16	1000	1	18	below detection	N
**-VE CONTROL 5**	na	na	na	control				0	below detection	Control
**GBR-15-7**	na	na	na	na	18.10.16	1400	1	18	below detection	N
**GBR-15-8**	21.0396	150.1081	error	na	18.10.16	1800	1	18	below detection	N
**GBR-15-9**	na	na	na	na	18.10.16	2200	1	18	below detection	N
**GBR-15-10**	na	na	na	na	19.10.16	0200	1	18	below detection	N
**GBR-15-11**	21.0292	150.1081	205.0071	ocean	19.10.16	0600	1	18	below detection	Y
**GBR-15-12**	21.0221	150.1135	272.8902	ocean	19.10.16	1000	1	18	below detection	Y
**GBR-16-1**	21.0151	150.1145	380.1455	ocean	20.10.16	0600	1	18	below detection	Y
**-VE CONTROL 6**	na	na	na	control				0	below detection	Control
**GBR-16-2**	na	na	na	na	20.10.16	1000	1	18	below detection	N
**GBR-16-3**	21.0187	150.1148	716.8459	ocean	20.10.16	1400	1	18	below detection	Y
**GBR-16-4**	21.0183	150.1139	541.7074	ocean	20.10.16	1800	1	18	below detection	Y
**GBR-16-5**	na	na	na	na	20.10.16	2200	1	18	below detection	N
**GBR-16-6**	na	na	na	na	21.10.16	0200	1	18	0.236	N
**GBR-16-7**	21.0173	150.1127	427.6637	ocean	21.10.16	0600	1	18	below detection	Y
**GBR-16-8**	21.0168	150.1131	389.6492	ocean	21.10.16	1000	1	18	below detection	Y
**-VE CONTROL 7**	na	na	na	control				0	below detection	Control
**GBR-16-9**	na	na	na	na	21.10.16	1400	1	18	0.147	N
**GBR-16-10**	na	na	na	na	21.10.16	1800	1	18	below detection	N
**GBR-16-11**	na	na	na	na	21.10.16	2200	1	18	below detection	N
**GBR-16-12**	21.7245	150.8378	271.5325	ocean	22.10.16	0200	1	18	below detection	Y

Figure S2. Faith's Phylogenetic Diversity (PD) for bacterial ASV originating from continental or oceanic derived air masses, with Chao1 diversity estimates shown for comparison. Phylogenetic diversity could not be performed with fungal ITS1 sequences due to the hypervariability of the region prohibiting the construction of a reliable phylogenetic tree.

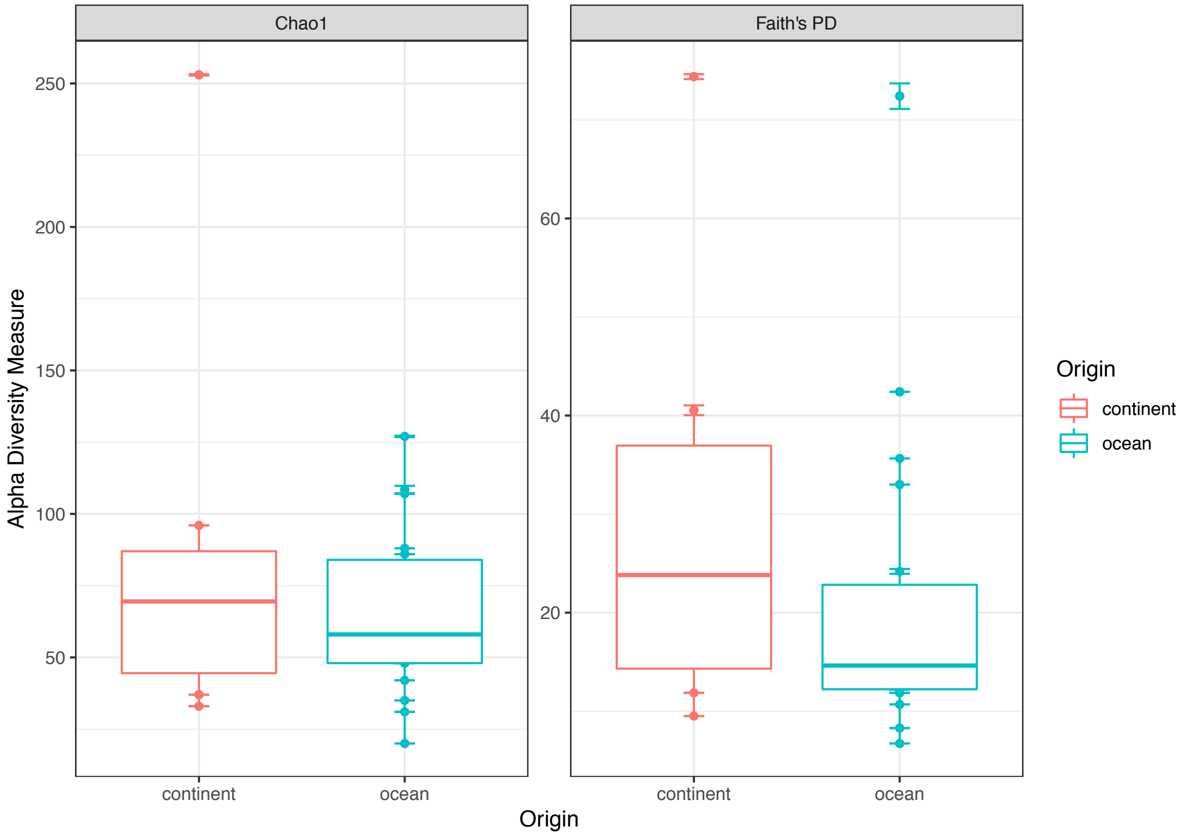

Figure S3. (a) Heatmap for 1,000 most abundant bacterial amplicon sequence variants (ASVs) by origin with control samples included (Continent n = 8, Ocean n = 19, control n = 7); (b) Heatmap for 1,000 most abundant fungal ASVs by origin with control samples included (Continent n = 8, Ocean n = 17, control n = 5). All samples were sequenced to near-asymptote. This analysis captured 99.98% of bacterial and 84.24% of fungal overall ASV-defined diversity.

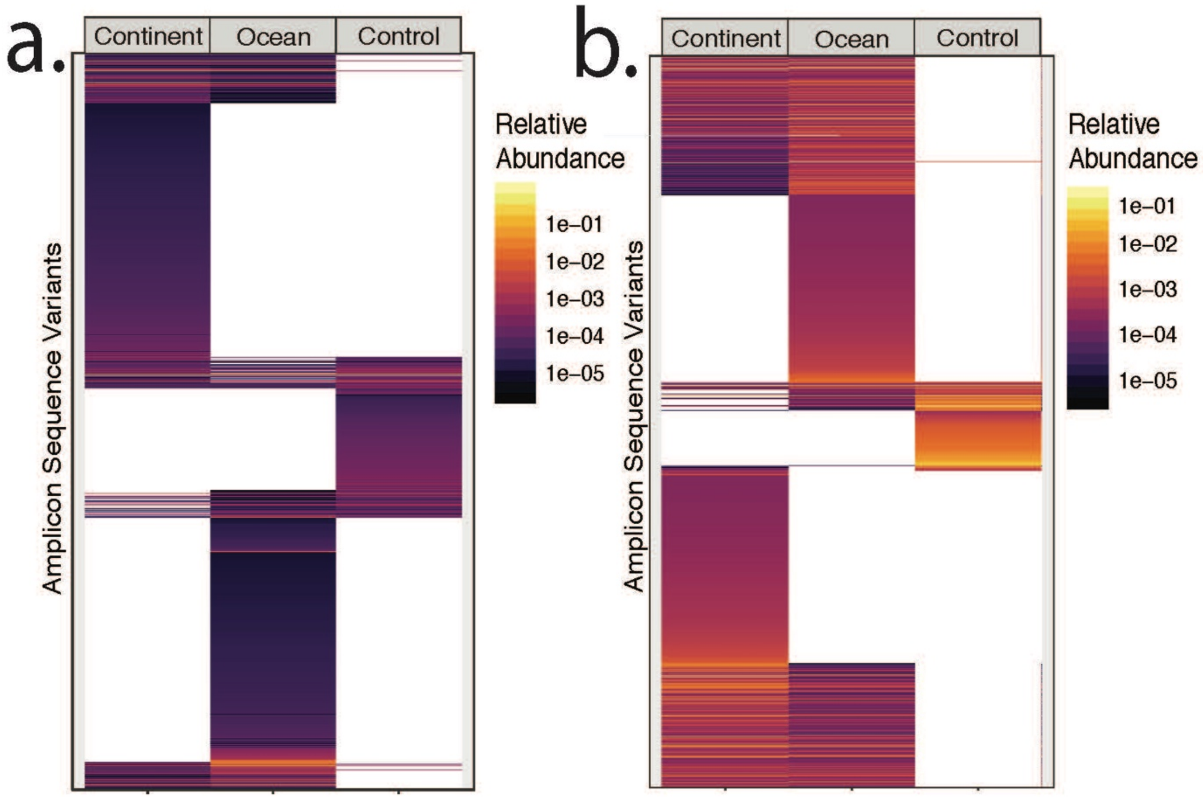

Figure S4. PCoA of Bray Curtis Dissimilarities for (a) bacteria and (b) fungi.

a.

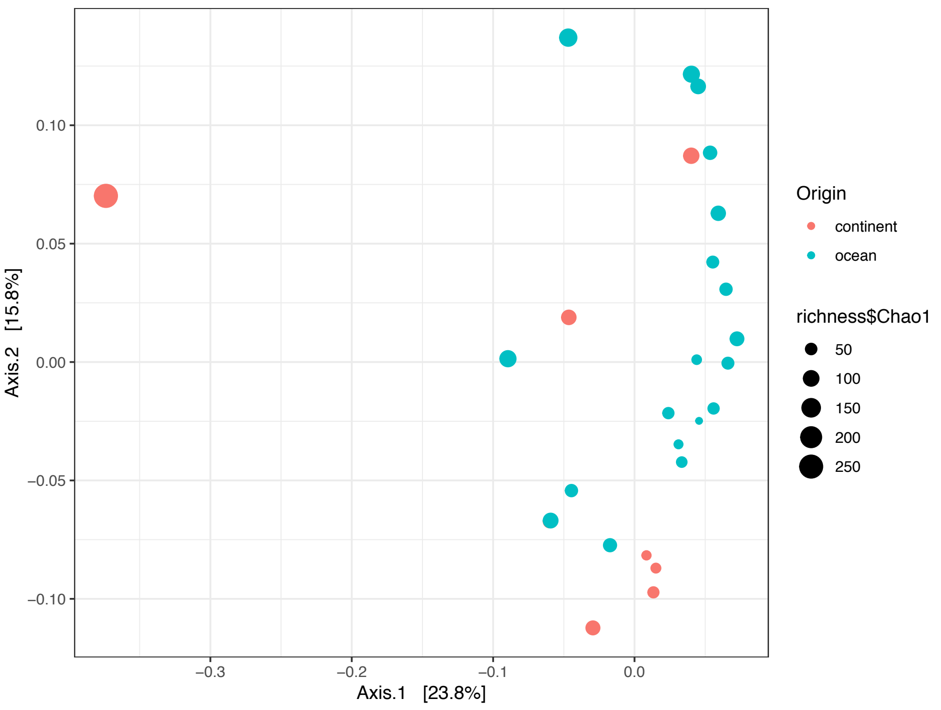

b.

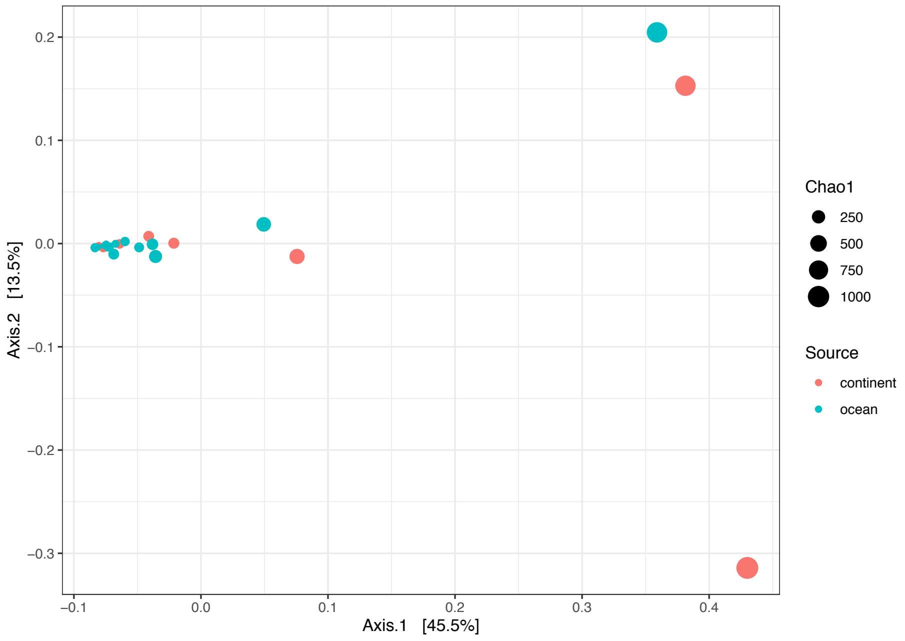

Figure S5. Linear distance decay using Kendall's rank correlation tau for (a) bacteria and (b) fungi.

a)

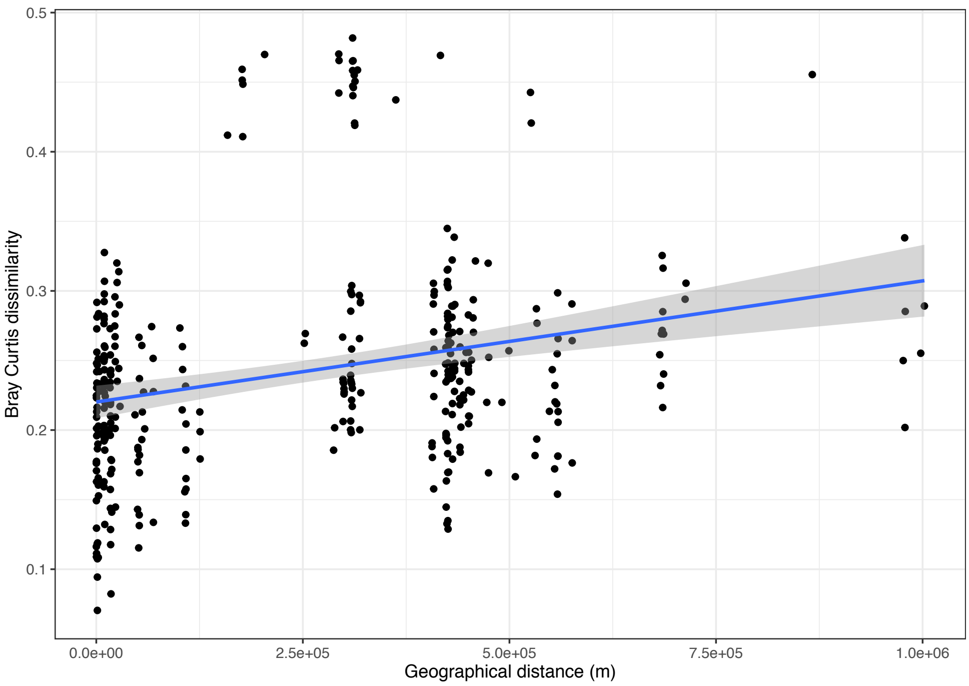

b)

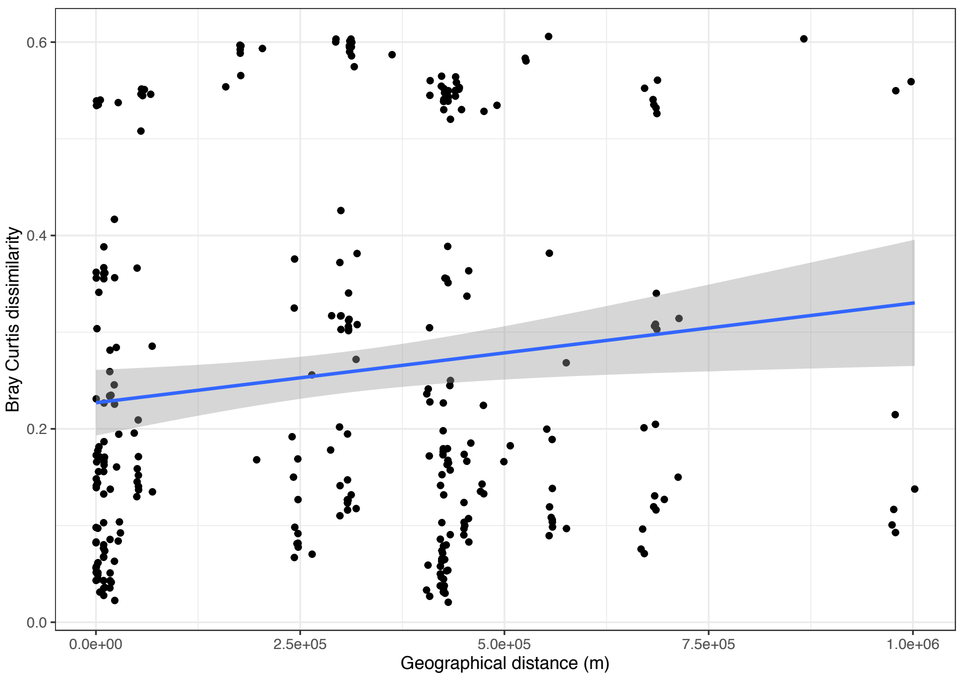

Figure S6. Relative abundance of bacterial taxa between air and coral sequence libraries for all taxa with >0.05% mean relative abundance. a) direct ASV matches between our study by source and the Pootakham et al coral microbiome [20], b) direct ASV matches that are classified at genus level between our study and the Pootakham et al coral microbiome [20] c) shared ASVs (by ≥97% sequence identity) between our study and the Wainwright et al coral microbiome [21].

.

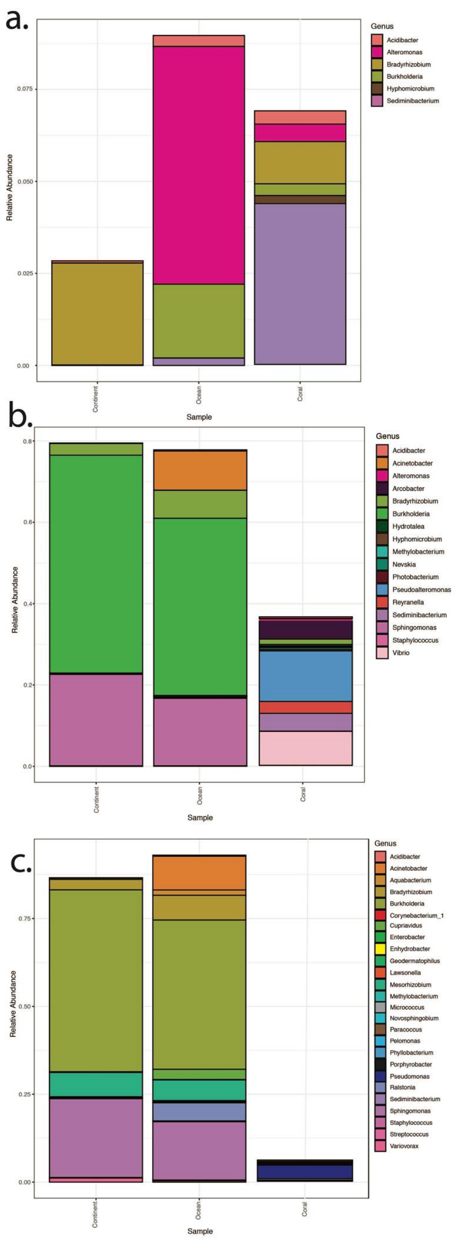

Table S2. Relative abundances of the 100 most abundant bacterial ASV's from genera identified in the literature as components of coral associated microbiomes**.**

Publication	Sample location	Microbiome type	Total number of matching genera	Matching genera	Abundance continent from matching genera	Abundance ocean matching genera
Angly et al.<br>(2016)<br>doi: 10.7717/peerj.1511	Great Barrier Reef	Water associated from reef surrounds	12	*Sphingomonas*<br>*Acinetobacter*<br>*Vibrio*<br>*Bacillus*<br>*Novosphingobium*<br>*Clostridium*<br>*Flavobacterium*<br>*Cellvibrio*<br>*Pseudoalteromonas*<br>*Pseudomonas*<br>*Lysobacter*<br>*Cupriavidus*	0.2255	0.2877
Bourne and Munn<br>(2005)<br>doi:10.1111/j.1462-2920.2005.00793.x	Great Barrier Reef	Coral associated	2	*Vibrio*<br>*Shewanella*	ND	ND
Cardinas et al.<br>(2011)<br>doi: 10.1038/ismej.2011.123	Caribbean - Colombia	Coral associated	9	*Vibrio*<br>*Pantoea*<br>*Pseudomonas*<br>*Stenotrophomonas*<br>*Paracoccus*<br>*Bacillus*<br>*Microbacterium*<br>*Brevibacterium*<br>*Micrococcus*	ND	ND
Sato et al.<br>(2009)<br>doi:10.1038/ismej.2009.103	Great barrier reef	Coral associated and pathogens	9	*Paracoccus*<br>*Sulfitobacter*<br>*Pseudoalteromonas*<br>*Bacillus*<br>*Clostridium*<br>*Desulfovibrio*<br>*Vibrio*<br>*Oscillatoria*<br>*Thalassospira*	ND	ND
Nelson et al.<br>(2011)<br>doi: 10.1038/ismej.2011.12	Moorea, French Polynesia	Water associated from reef surrounds	6	*Acinetobacter*<br>*Pseudomonas*<br>*Pseudoalteromonas*<br>*Paracoccus*<br>*Bacillus*<br>*Pirellula*	ND	0.0941
Ainsworth et al.<br>(2015)<br>doi:10.1038/ismej.2015.39	Great barrier reef and Hawaii	Coral associated - core microbiome	13	*Ralstonia*<br>*Bacteroides*<br>*Pseudomonas*<br>*Pseudoalteromonas*<br>*Corynebacterium*<br>*Limnobacter*<br>*Streptococcus*<br>*Halomonas*<br>*Staphylococcus*<br>*Acinetobacter*<br>*Stenotrophomonas*<br>*Vibrio*<br>*Psychrobacter*	0.0029	0.1466

Ziegler et al. (2017) doi: 10.1038/ncomms14213	Ofu Island, American Samoa	Coral associated	5	*Vibrio* *Pseudomonas* *Pseudoalteromonas* *Brachybacterium* *Arcobacter*	ND	ND
Hernandez-Agreda et al. (2016) doi: 10.1128/mBio.00560-16	Great Barrier Reef and Coral Sea	Coral associated - core microbiome	9	*Pseudomonas* *Mycobacterium* *Vibrio* *Alcanivorax* *Staphylococcus* *Ralstonia* *Pseudoalteromonas* *Acinetobacter* *Corynebacterium*	0.0024	0.1459
**Mean match**					**2.92%**	**8.44%**

ND = not detected.

Table S3. Relative abundance of the most abundant fungal ASV's at genus level to genera identified from the literature as components of coral associated microbiomes**.**

**Publication** (see Supplementary Methods references list)	**Sample location**	**Microbiome type**	**Total number of matching genera**	**List of matching genera**	**Matching genera to continent air**	**Matching genera to ocean air**
Littman et al. (2011)	Great Barrier Reef	Coral associated	1	*Gibberella*	0.0021	ND
Morrison-Gardiner (2002)	Australian tropical waters	Sediment and marine organism related	6	*Alternaria* *Cladosporium* *Curvalaria* *Epicoccum* *Nigrospora* *Periconia*	0.1825	0.2577
Sweet at al. (2013)	Caribbean	Coral associated and pathogens	0		N/A	
Staley et al. (2017)	Caribbean	Coral associated and water associated proximate to reef	1	*Exophiala*	ND	0.0042
**Mean score**					**6.16%**	**8.73%**

ND = not detected.
